# Supplementary material for: A novel frameshift mutation in Phosphoinositide 3-kinase regulatory subunit 1 (PIK3R1) causes immunodeficiency and Amyotrophic Lateral Sclerosis (ALS)
Source: bioRxiv. 2025 May 28:2025.05.23.655625. Preprint. [Version 1] doi: 10.1101/2025.05.23.655625 (PMC12190176; doi:10.1101/2025.05.23.655625)
Supplement: Supplement 1 — Supplemental Figure 1. Peripheral blood immunophenotype and CSF findings in a patient with a PIK3R1 c.1710dup mutation. A) Peripheral blood immunophenotyping showing normal counts of CD4+ T cells, CD8+ T cells, and CD19+ B cells. B) CSF analysis demonstrates intermittent lymphocytic pleocytosis, elevated IgG levels, and isolated OCBs. Supplemental Figure 2. The effect of PIK3R1 c.1710dup mutation on AKT signaling. Immunoblot analysis of pAKT (Thr308), pAKT (Ser473), total AKT from patient’s iMNs lysate compared to edited and healthy controls are shown. A) Immunoblots from iMNs in patient nd healthy are shown. Densitometry data from pooled three independent experiments confirmed elevated pAKT (Thr308 and Ser473)/Total AKT ratio in patient’s iMNs compared to healthy controls. B) Pooled densitometry data from 4 independent immunoblot experiments of phospho-S6 and total S6 showed patient derived iMNs have decrease pS6 to Total S6 ratio compared to healthy cells. Supplemental Figure 3. scRNA-seq analysis of iMNs with PIK3R1 c.1710dup mutation A) Variable quality control metrics across scRNA-seq data from iMNs. B) Marker gene expression dot plots of all iMN scRNA-seq samples. C) Proportion bar plot showing percentage of cells in each cluster for the patient iMN and matching control. Supplemental Figure 4. scRNA-seq analysis of brain organoids with PIK3R1 c.1710dup mutation. A) Heatmaps show differentially expressed genes for neuronal and B) glial-like cell types. For visualization, genes with an adjusted p-value less than 0.1 were selected and the apoptotic pathway genes are annotated. Supplemental Table 1. Antibodies for immunophenotyping of CSF using spectral flow cytometry are shown. [file media-1.pdf]

Figure 1 Supp

Peripheral Blood

	Ref. Value	07/2021	02/2017	6/2016	3/2016	1/2016	10/2015	02/2013
**Medication**		Rituximab discontinued				Rituximab started		
**Blood**								
**CD3#**	714-2266/mcL	1196	1062	1005	1236	1282	889	1292
**CD4/CD3#**	359-1565/mcL	383	392	331	407	291	225	356
**CD8/CD3#**	178-853/mcL	728	597	603	750	894	577	815
**CD19+#**	61-321/mcL	30	1	25	0	116	-	-
**NK Cell#**	126-729/mcL	63	35	25	37	65	42	58
**DNT Cell%**	1.3-9.2%						8.3	7.9
**CD4/CD62L+/CD45RA+/CD3%**	7.6-37.7%						1.9	
**CD3/CD4/CD62L+/CD45RA-%**	10.4-30.7%						16.7	
**CD3/CD4/CD62L-/CD45RA-%**	2.3-15.6%						3.7	
**CD4/CD62L-/CD45RA+/CD3%**	0.0-1.5%						0	
**CD8/CD62L+/CD45RA+/CD3%**	5.7-19.7%						20.7	
**CD3/CD8/CD62L+/CD45RA-%**	1.5-10.3%						17.6	
**CD3/CD8/CD62L+/CD45RA-%**	1.1-9.2%						10.7	
**CD8/CD62L-/CD45RA+/CD3%**	0.7-7.8%						8	
**CD20%**	3.0-19.0%						7.8	

CSF

	Ref. Value	07/2021	02/2017	6/2016	3/2016	1/2016	10/2015	02/2013
**Medication**		Rituximab discontinued				Rituximab started		
**CSF**								
**Glucose**	40-70 mg/dL	59	50	50	51	49	65	49
**Protein**	15.0-40.0 mg/dL	34	42	35	36	29	30	41
**WBC Count**		3	11	9	8	7	0	3
**Lymphocyte %**		--	92	94	92	96	--	--
**Oligoclonal Bands**		1 CSF, 0 serum	0 CSF, 0 serum	CSF bands only	0 CSF, 0 serum	0 CSF, 0 serum	CSF bands only	CSF bands only
**IgG Index**	0.26-0.62 ratio	0.33	0.4	0.35	0.55	0.45	0.54	0.58

Figure 2 Supp

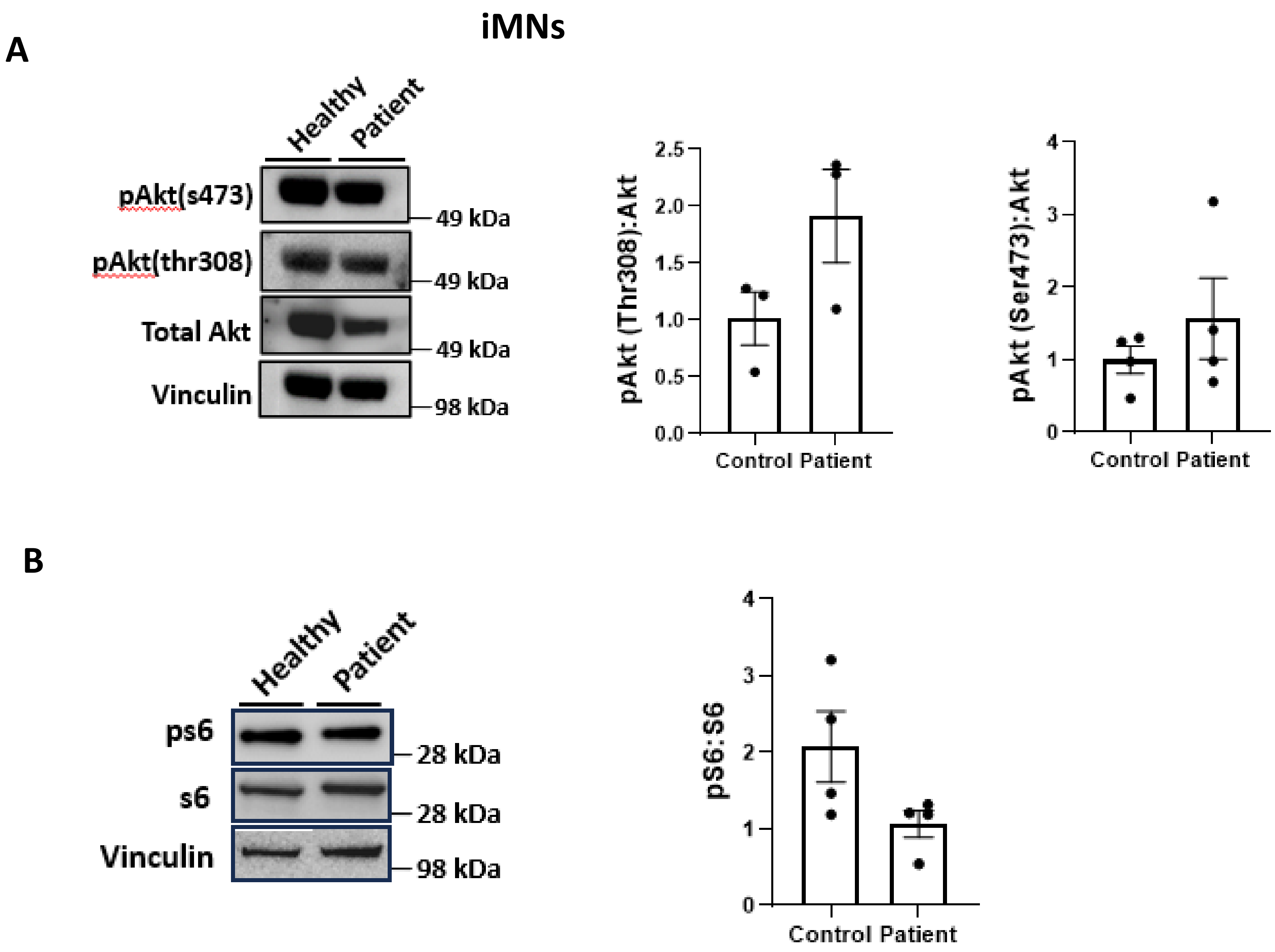

Figure 3 Supp

A

	Number of reads	Estimated cell count	Mean reads per cell	Median genes per cell
**Mutated**	811,218,958	11,000	73,747	5,596
**Control**	878,462,975	13,700	64,121	5,391
**Patient**	460,223,516	4,026	114,313	2,220
**Healthy**	1,189,668,728	14,785	80,465	5,790

B

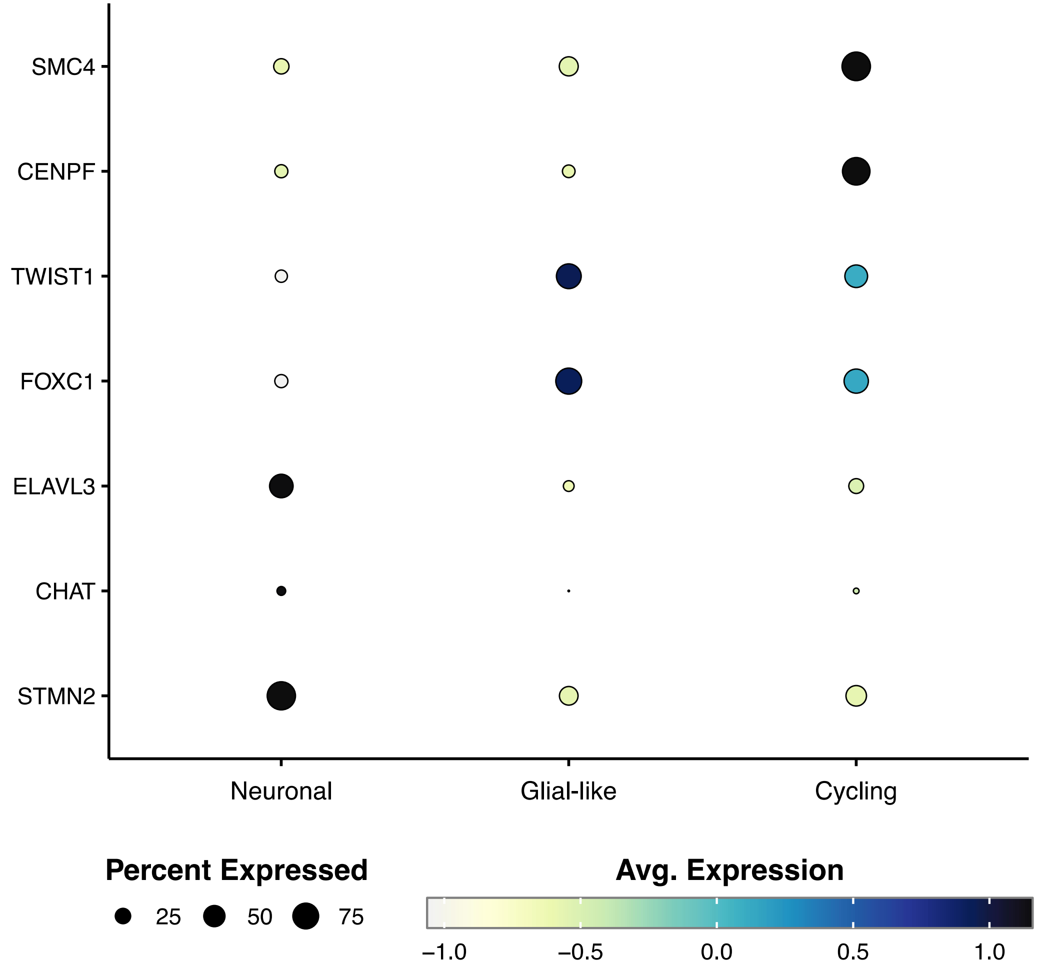

C

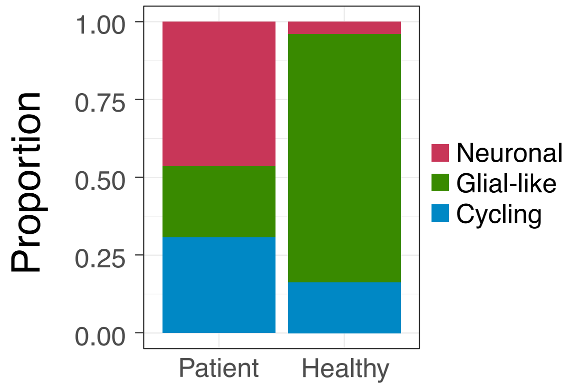

Figure 4 Supp

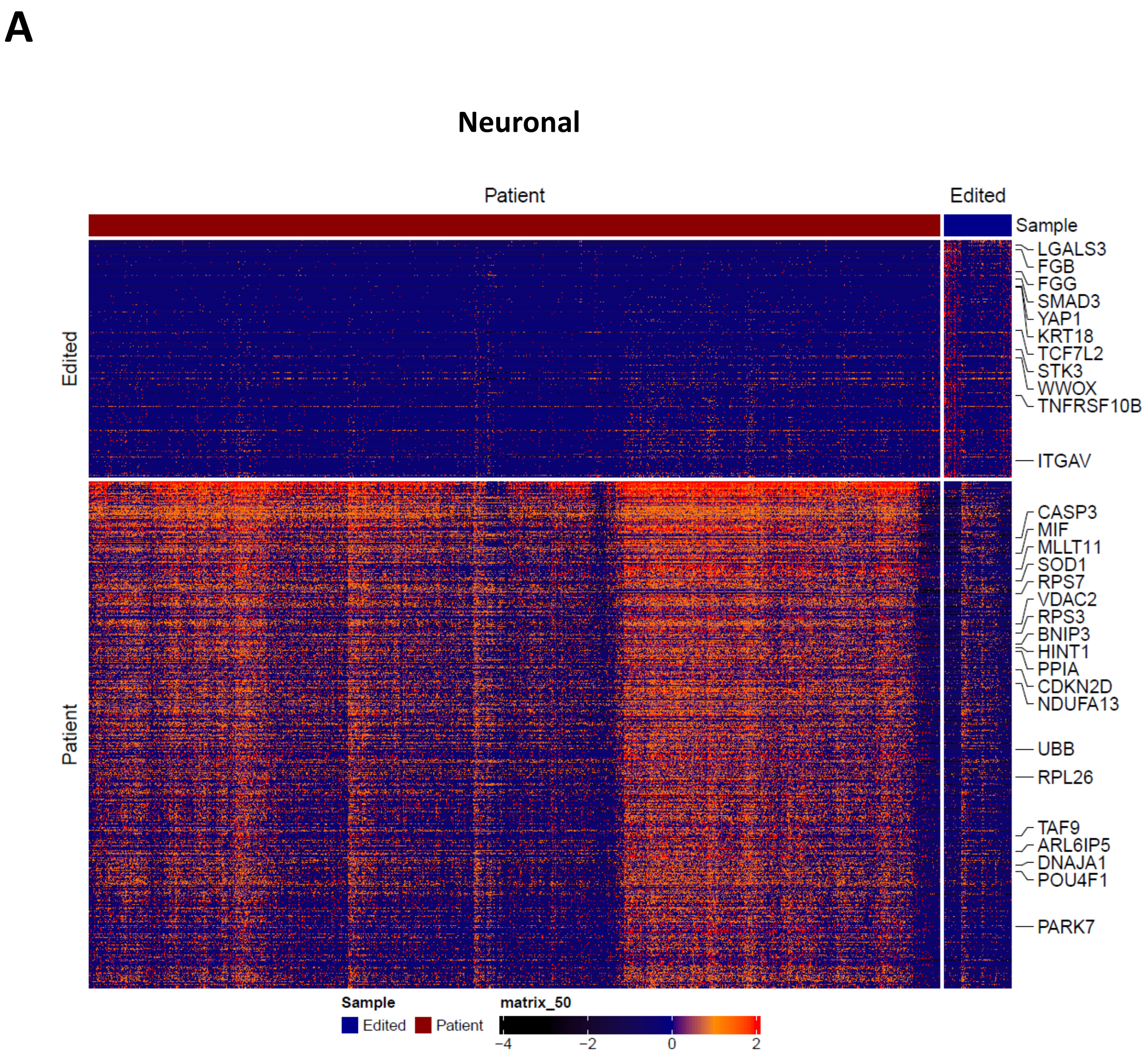

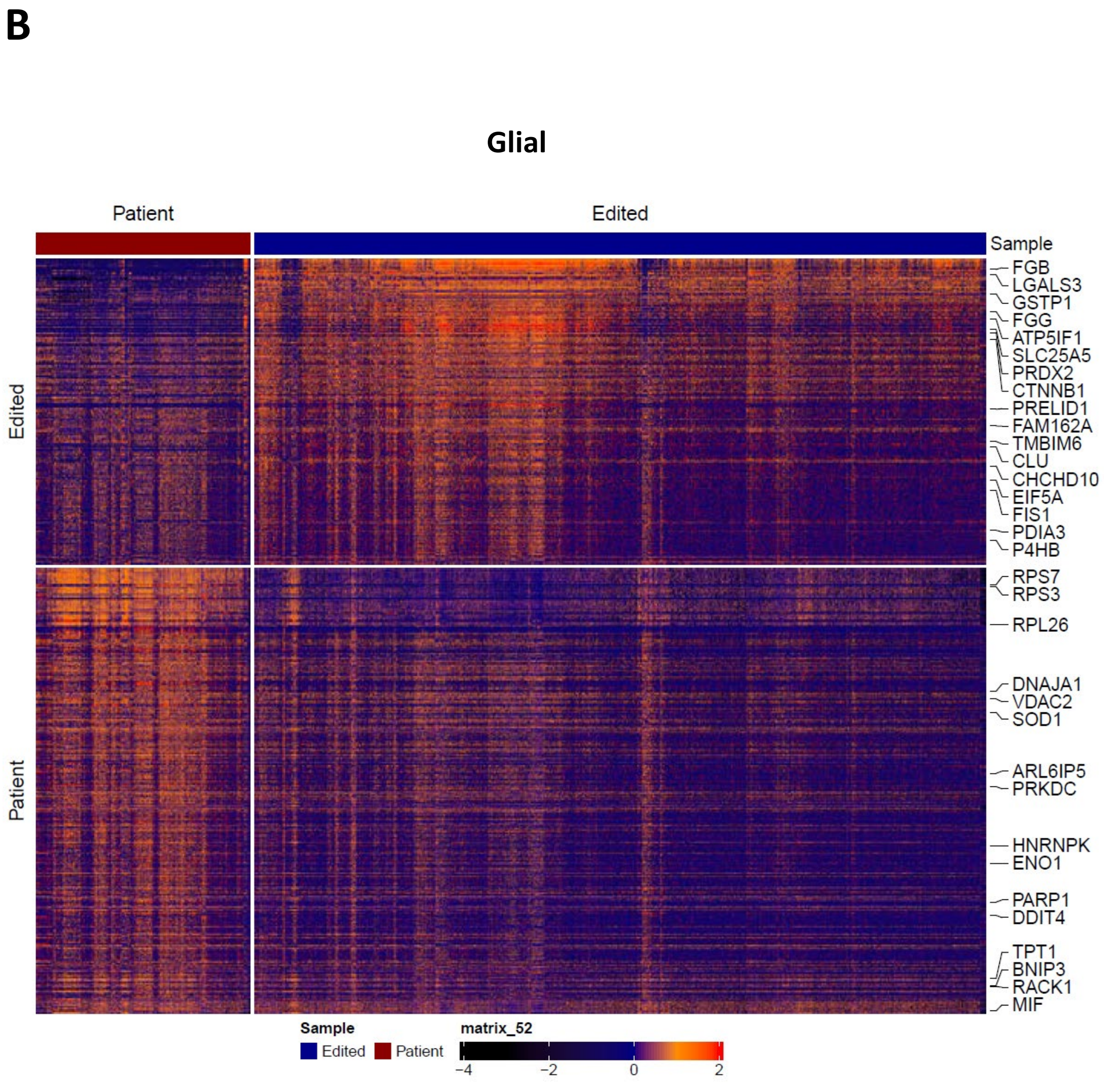

Table 1 Supp

Marker	Clone	Fluorophore	Supplier	Cat. #
CD45	2D1	PerCP	BioLegend	368505
CD8	3B5	Qdot 800	Thermo Scientific	Q22157
CD16	3G8	BUV496	BD	612944
CD45RA	5H9	BUV395	BD	740315
CD123	6H6	Super Bright 436	Thermo Scientific	62-1239-42
CD335	9E2	PE	BioLegend	331908
TCR γδ	B1.1	PerCP-eFluor 710	Thermo Scientific	46-9959-42
CD11c	B-Ly6	BUV661	BD	612967
CD28	CD28.2	BV605	BioLegend	302968
CD19		spark NIR 685	Biolegend	302270
CD4	SK3	cFlour YG584	Cytek	R7-20041-100T
CD95	DX2	PE-Cy5	BioLegend	305610
PD-1	EH12.2H7	BV421	BioLegend	329920
CD86	FUN-1	BB515	BD	564544
CXCR3	G025H7	BV650	BioLegend	353730
CCR6	G034E3	BV711	BioLegend	353436
CCR7	G043H7	BV785	BioLegend	353230
CD20	HI47	Pacific Orange	Thermo Scientific	MHCD2030
CD127	HIL-7R-M21	APC-R700	BD	565185
CD38	HIT2	APC-eFluor 780	Thermo Scientific	47-0389-41
CD57	HNK-1	FITC	BioLegend	359604
CD161	HP-3G10	eFluor 450	Thermo Scientific	48-1619-41
IgD	IA6-2	BV480	BD	566138
CD11b	ICRF44	PerCP-Cy5.5	BioLegend	301328
HLA-DR	L243	BV570	BioLegend	307637
CD25	M-A251	PE-Cy7	BioLegend	356108
CD24	ML5	PE/Dazzle594	BioLegend	311134
CD27	M-T271	APC	BioLegend	356410
CD14	MφP9	BUV563	BD	741441
Viability	N/A	LIVE DEAD Blue	Thermo Scientific	L23105
CD56	NCAM16.2	BUV737	BD	564447
CD3	OKT3	BV510	BioLegend	317332
CD33	P67.6	Alexa Fluor 647	BioLegend	366626
CXCR5	RF8B2	BV750	BD	747111
CD45RO	UCHL1	BUV805	BD	748367
